# Comparison of Efficacy of Anti-interleukin-17 in the Treatment of Psoriasis Between Caucasians and Asians: A Systematic Review and Meta-Analysis

**DOI:** 10.3389/fmed.2021.814938

**Published:** 2022-01-25

**Authors:** Danyi Zhang, Jianing Qiu, Xing Liao, Yi Xiao, Minxue Shen, Yaxiong Deng, Danrong Jing

**Affiliations:** ^1^Department of Dermatology, Xiangya Hospital, Central South University, Changsha, China; ^2^Xiangya School of Medicine, Central South University, Changsha, China; ^3^National Clinical Research Center for Geriatric Disorders, Xiangya Hospital, Central South University, Changsha, China; ^4^Department of Social Medicine, Xiangya School of Public Health, Central South University, Changsha, China; ^5^Department of Dermatology, The Second Xiangya Hospital, Central South University, Changsha, China; ^6^Immunology Section, Lund University, Lund, Sweden

**Keywords:** psoriasis, secukinumab, brodalumab, ixekizumab, Asian, Caucasian

## Abstract

**Background:**

Interleukin-17 (IL-17) monoclonal antibody drugs have been increasingly significant in the treatment of psoriasis, but it is not clear whether the efficacy is equivalent across ethnicities.

**Objective:**

To explore the differences of short-term efficacy of IL-17 inhibitors between Caucasians and Asians.

**Methods:**

The pooled log risk ratio (logRR) between the groups was estimated. The meta-regression analysis on the logRR was performed, with the proportion of Caucasian patients as the covariate. The subgroup analysis was performed by specific IL-17 inhibitors.

**Results:**

Of the 1,569 potentially relevant studies, sixteen randomized controlled trials (RCTs) were included. For the Psoriasis Area and Severity Index 75 (PASI 75) response at week 12, the pooled logRR of the Asian group and the Caucasian group was 2.81 (95% CI: 2.27–3.35, *p* < 0.001) and 2.93 (95% CI: 2.71–3.16, *p* < 0.001), respectively, indicating no significant difference of efficacy between Asians and Caucasians. The meta-regression analysis did not show an association of the proportion of Caucasians with the effect size (β = 0.3203, *p* = 0.334). In the subgroup analysis, the comparison results of secukinumab were consistent with the main analysis.

**Limitations:**

Only the short-term efficacy was explored. The data from Asian countries were limited.

**Conclusions:**

The short-term efficacy of IL-17 inhibitors in the treatment of psoriasis has no significant difference between Caucasians and Asians.

**Systematic Review Registration:**

PROSPERO, identifier CRD42020201994, https://www.crd.york.ac.uk/prospero/.

## Introduction

Psoriasis is a chronic inflammatory disease driven by proinflammatory cytokines (1), affecting 2–3% of the population of the world (2). Although psoriasis presents in all the ethnic groups, there are variances in the incidence that Asian countries are significantly lower than European countries (3–5). The psoriasis phenotype is also different among ethnicities. Chronic plaque psoriasis is the most common form of psoriasis, accounting for about 90% of cases (6). Patients with psoriasis in western countries are more likely to present large plaque psoriasis, while small and intermediate plaque psoriasis is more common in Asian psoriasis patients (7, 8). Although the prevalence of moderate-to-severe psoriasis is relatively low in Asia, its severity is significantly higher than in Caucasians (9).

At present, interleukin-17 (IL-17) pathway antagonists in the market are mainly secukinumab (AIN457), ixekizumab (LY2439821), and brodalumab (AMG827), which were approved by the US Food and Drug Administration (FDA) in 2015, 2016, and 2017, respectively, for the treatment of moderate-to-severe psoriasis (10, 11). Secukinumab and ixekizumab are human monoclonal antibodies against IL-17A, while brodalumab antagonizes the IL-17A receptor (IL-17RA) and disrupts the signal transduction of IL-17A, IL-17C, (12) IL-17F, and IL-17A/F heterodimer (13).

In previous studies, three IL-17 inhibitors have shown excellent efficacy in regional clinical trials (14–16). However, a study showed that molecular phenotypes of small (Asian) and large (Western) plaque psoriasis show co-activation of genes in the IL-17 pathway, while with different regulatory gene sets. IL-17A and IL-17-regulated proinflammatory cytokines were highly expressed in Asian small plaque psoriasis, but lower in Western large plaque psoriasis (7). Moreover, one study suggested better clinical efficacy of secukinumab, brodalumab, and ixekizumab in the Japanese groups compared to Western subjects (17). We hypothesize that there may be a difference in the efficacy of IL-17 inhibitors between Asians and Caucasians. This study aims to integrate and analyze the efficacy of IL-17 inhibitors on psoriasis from randomized controlled trials (RCTs) obtained by systematic retrieval and strict screening to explore the differential efficacy between Asian and Caucasian patients.

## Methods

### Data Searches and Sources

The systematic literature search was conducted in the PubMed, Embase, and Cochrane databases, the Wanfang Database, and the Chinese National Knowledge Infrastructure Data of Chinese Journals from inception up to November 29, 2020. No restrictions by language were employed. The analysis included the full-study population of the randomized, double-blind, and placebo-controlled trials of secukinumab, brodalumab, and ixekizumab. We also reviewed abstracts and presentations from all the major conference proceedings.

The keywords used in the search strategy included “psoriasis or psoriatic,” “secukinumab or cosentyx,” “brodalumab or siliq,” “ixekizumab or taltz,” and “randomized controlled trials.” The search strategy adjusted to a controlled vocabulary for each database. The full search terms and strategies are given in Supplementary File 1.

This study was registered on the PROSPERO (registration number #CRD42020201994). Changes from the protocol were mentioned in supplementary methods.

### Inclusion and Exclusion Criteria

The inclusion criteria for this systematic review and meta-analysis were: (1) patients: moderate-to-severe plaque psoriasis; (2) intervention: IL-17 antagonists (secukinumab, brodalumab, and ixekizumab); (3) comparator: placebo; (4) outcomes: 75% or greater and 90% or greater improvement in the Psoriasis Area and Severity Index score from baseline (PASI 75 and PASI 90) as primary outcome; and (5) study design: double-blind, randomized placebo-controlled trials.

We excluded: (1) nonrandomized placebo-controlled trials; (2) observational studies, case reports/series, or review articles; (3) studies that did not provide the proportion of Caucasian patients; and (4) outcome data could not be extracted.

### Study Selection

Titles and/or abstracts of all the relevant studies were screened independently by two reviewers (DZ and JQ) to identify studies that met the above inclusion criteria. The full text of these potentially eligible studies was retrieved and independently assessed for eligibility by two investigators. Any disagreement between the two reviewers regarding the eligibility of a study was resolved through discussion with a third reviewer (XL).

### Data Extraction

The extracted data included the following: the name of the author; year of publication; the characteristics of the study population (including number, sex ratio, the races or the Caucasian proportion, the average of age and body mass index (BMI), the baseline PASI, and previous therapy history); dosing schedule; week of evaluation of response; and study outcomes at the endpoint (including PASI 75, PASI 90, sPGA0/1, IGA0/1, and DLQI0/1 response rates). We also collected the incidence of adverse event (AE), serious AE (SAE), discontinuations due to AE, and some most common adverse effects (e.g., infections, nasopharyngitis, headache, and upper respiratory tract infection).

Two reviewers (DZ and JQ) extracted data independently; discrepancies were identified and resolved through discussion [with a third reviewer (XL) when necessary].

### Quality Assessment

To assess the risk of bias within each included study, we used the Cochrane risk-of-bias tool, which includes the following domains: sequence generation (selection bias), allocation sequence concealment (selection bias), blinding of participants and personnel (performance bias), blinding of outcome assessment (detection bias), incomplete outcome data (attrition bias), selective outcome reporting (reporting bias), and other potential sources of bias. The judgment of authors is categorized as “low risk,” “high risk,” or “unclear risk” of bias. We also used the Grading of Recommendation, Assessment, Development, and Evaluation (GRADE) system to assess the quality of evidence for every outcome. Two independent reviewers assessed eligibility criteria and extracted data and any disagreements were resolved by discussion.

### Statistical Analysis

Extracted data were combined for the meta-analysis using the STATA version 16.0 (StataCorp LLC, 4905 Lakeway Drive, College Station, Texas, USA). We chose the PASI 75 response at week 12 as the primary outcome. Dichotomous data were pooled as the log risk ratios (logRRs) with the respective 95% CIs using the Mantel–Haenszel fixed effects method. The data of ethnically Asian patients and the Caucasian patients were classified and pooled separately. The meta-regression analysis was performed to investigate the association of the basal PASI and proportion of Caucasian patients with the efficacy, as the basal PASI seemed to be slightly higher in the Asian group and some studies included a variety of ethnicities (but still Caucasian predominant). The subgroup analysis was performed by specific agents and doses. Sensitivity analysis was conducted to examine whether one or more studies deviated from the overall results. Heterogeneity between studies was assessed using the *Q*-statistic (significance level at *p* = 0.050) and *I*^2^ statistic (significant heterogeneity, *I*^2^ > 50%; insignificant heterogeneity, *I*^2^ < 40%). A *Z*-test was performed to assess the combined statistical outcomes. The funnel plot analysis was used for the detection of potential publication bias.

## Results

### Study Selection

**Figure 1:**
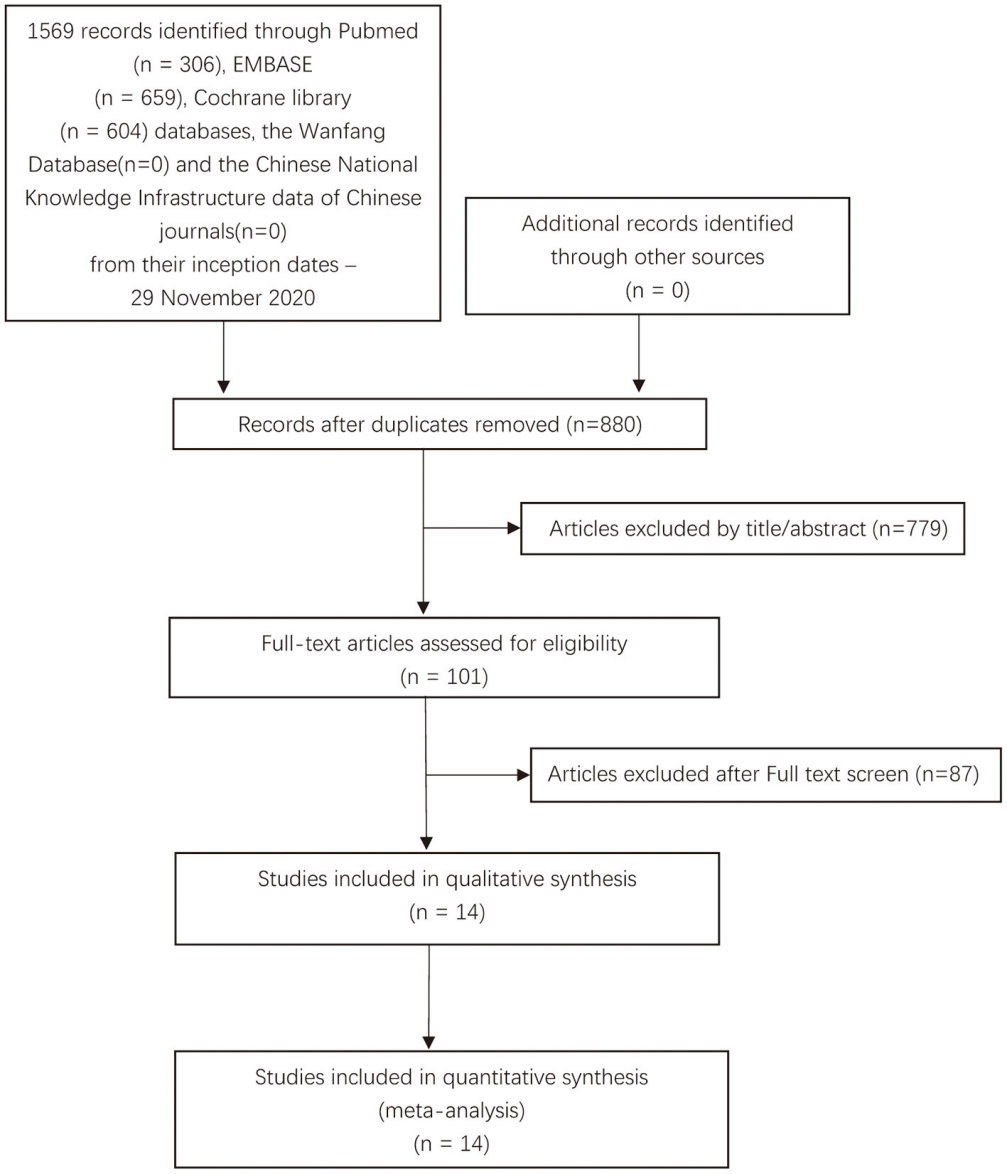
The Preferred Reporting Items for Systematic Reviews and Meta-Analyses (PRISMA) search strategy for the present studies.

Our search identified 1,569 records through database search: PubMed (*n* = 306), Embase (*n* = 659), Cochrane library (*n* = 604) databases, the Wanfang Database (*n* = 0), and the Chinese National Knowledge Infrastructure data of Chinese journals (*n* = 0). After removing duplicates, 880 records were screened and 779 were excluded (which were not RCTs, not related to psoriasis, or not related to IL-17 antagonists, etc.). After a full-text review of the remaining 101 references, we excluded 87 studies according to the exclusion criteria. Finally, 14 studies with a total of 16 double-blind, randomized placebo-controlled trials that met the inclusion criteria were included in this meta-analysis (Figure 1) (18–31).

### Study Characteristics

In total, 6,765 patients (4,843 patients with IL-17 antagonists and 1,922 patients with placebo) were involved in this study. Of the 14 studies included, ten studies were phase III and four studies were phase II. Among them, six studies specifically recruited Asian patients (Japanese, Indian, or Chinese) including four studies for secukinumab, one study for brodalumab, and one study for ixekizumab. Eight studies predominantly recruited Caucasian patients including four studies for secukinumab, two studies for brodalumab, and two studies for ixekizumab. All the included studies had at least the two intervention groups, and the one placebo group and reported the PASI 75, from baseline at week 12. All the studies involved in this meta-analysis shared similar baseline characteristics and inclusion criteria. Fourteen studies reported the average age, the baseline PASI score, and prior treatment history and nine studies reported averaged BMI (Supplementary File 2).

### Risk-of-Bias Assessment

The risk-of-bias among the included studies was rated as “low risk,” “unclear risk,” or “high risk” (Supplementary File 3; Supplementary Figures 1, 2). 10 studies (62.5%) reported an adequate randomization method of all the sixteen studies, while allocation concealment was sufficient in 8 studies (50%). In all of these studies, the blinding of participants and personnel was ensured. The risk of attrition bias in 1 (6.25%) trial was unclear and in another trial was high. The risk of reporting bias was low in all of these studies. Two (12.5%) studies published a high risk of other bias. The funnel plot showed slight asymmetry (Supplementary File 3; Supplementary Figure 3), but Egger's test (β = 0.511, *p* = 0.522) and Begg's test (score = 22.211, *p* = 0.685) indicate no publication bias.

### Main Analysis

**Figure 2:**
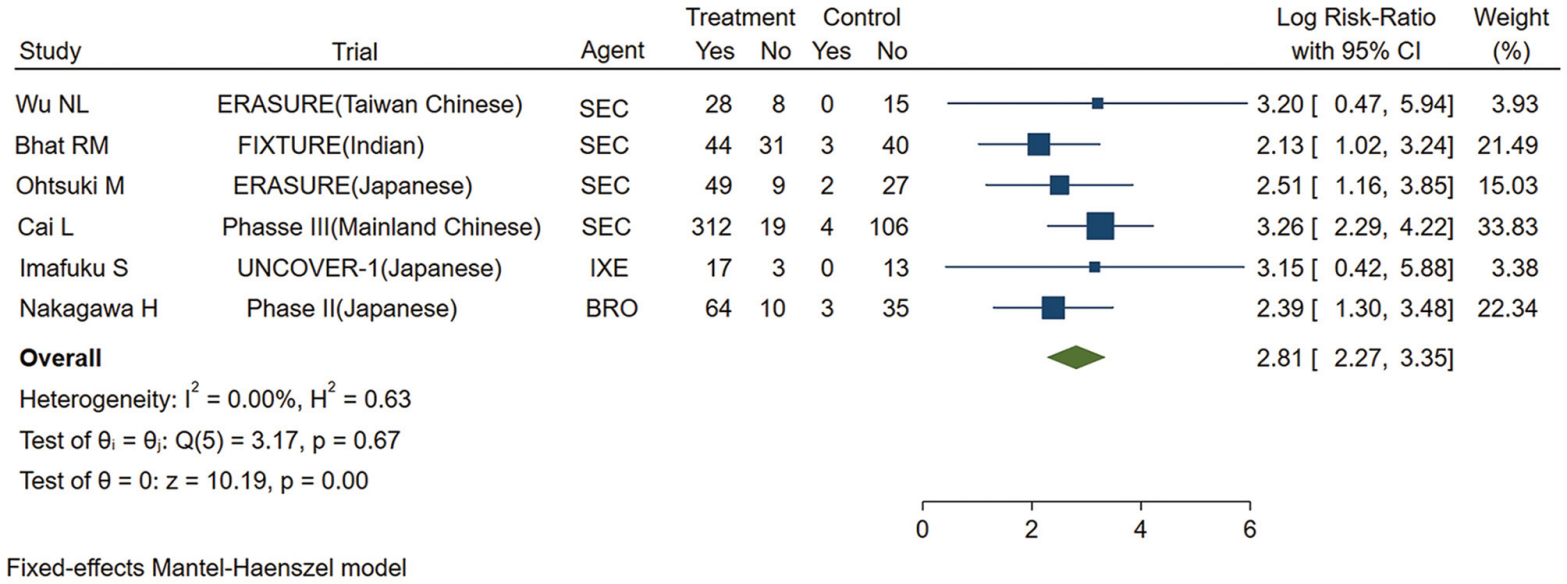
Meta-analysis of Asian patients treated with IL-17 inhibitors or placebo for the PASI 75 response at week 12. RR, risk ratio; SEC, secukinumab; BRO, brodalumab; IXE, ixekinumab; Q4W, every 4 weeks; Q2W, every 2 weeks; IL-17, interleukin-17; PASI, Psoriasis Area and Severity Index 75.

**Figure 3:**
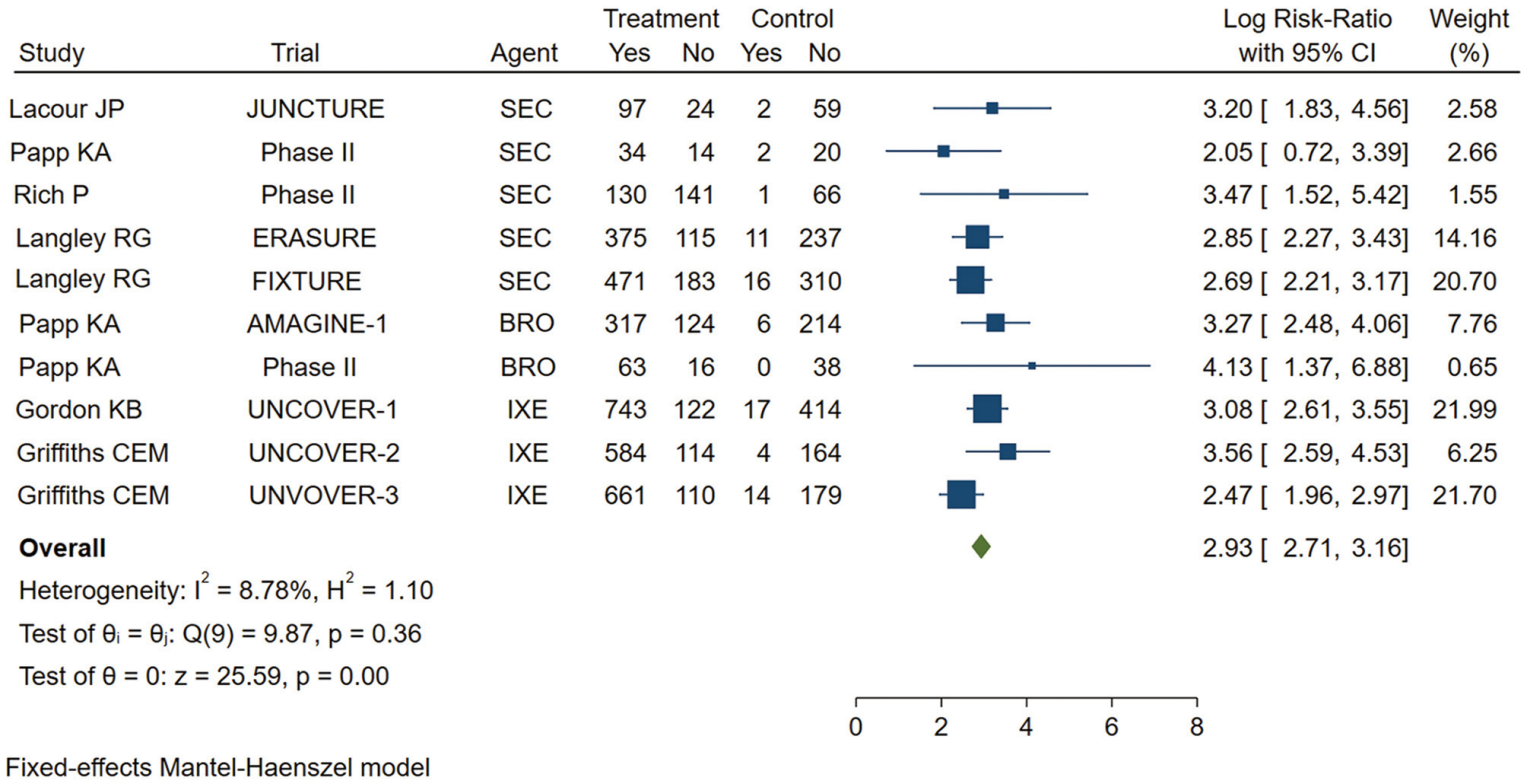
Meta-analysis of Caucasian patients treated with IL-17 inhibitors or placebo for the PASI 75 response at week 12. RR, risk ratio; SEC, secukinumab; BRO, brodalumab; IXE, ixekinumab; Q4W, every 4 weeks; Q2W, every 2 weeks; IL-17, interleukin-17; PASI, Psoriasis Area and Severity Index 75.

As shown in Figure 2, the pooled estimate of the Asian group favored IL-17 inhibitors over placebo for the PASI 75 response at week 12 (logRR = 2.81,95% CI: 2.27 to 3.35, *p* < 0.001). As shown in Figure 3, the pooled estimate of the Caucasian group also favored IL-17 inhibitors over placebo for the PASI 75 response at week 12 (logRR = 2.93, 95% CI: 2.71 to 3.16, *p* < 0.001). The results showed that there is no significant difference in the efficacy between the groups, as the 95% CIs overlapped. Evidence quality was evaluated on each outcome index. The results of the GRADE evaluation showed that both the Asian group PASI 75 and the Caucasian group PASI 75 outcome the quality of evidence were high (Table 1).

**Table 1:** Quality of evidence in included systematic reviews with the Grading of Recommendation, Assessment, Development, and Evaluation (GRADE).

**Research Indicators**	**Risk of bias**	**Inconsistency**	**Indirectness**	**Imprecision**	**Publication bias**	**Included RCTs (patients)**	**Quality of evidence**
PASI 75 at week 12 (Asian group)	Not serious	Not serious	Not serious	Not serious	Not serious	5(402)	High
PASI 75 at week 12 (Caucasian group)	Not serious	Not serious	Not serious	Not serious	Not serious	10(6,212)	High

### Meta-Regression Analysis

**Figure 4:**
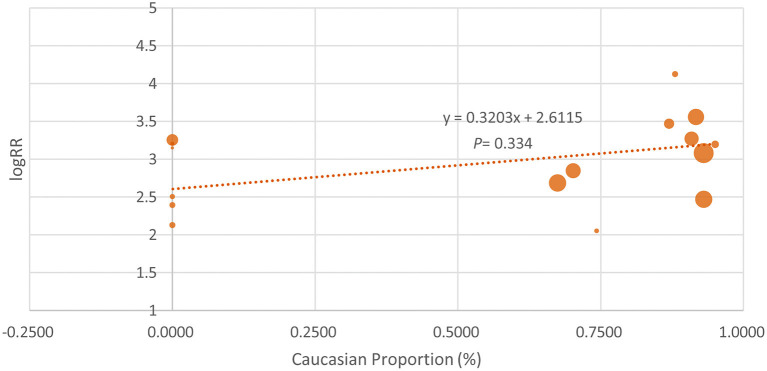
The meta-regression analysis investigating the association of Caucasian proportion and the log risk ratio (logRR) treatment effect across studies (the PASI 75 response at week 12).

**Figure 5:**
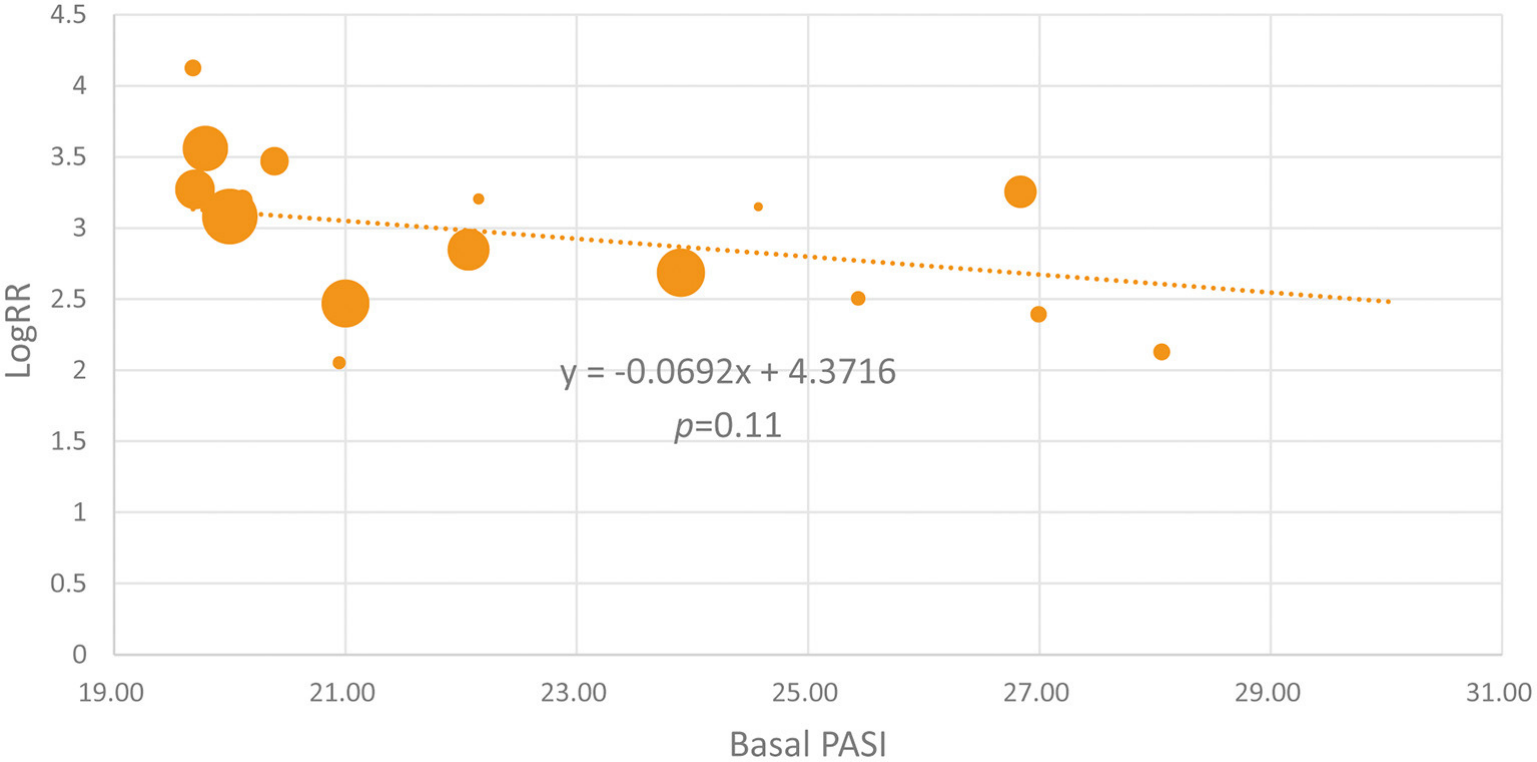
The meta-regression analysis investigating the association of the basal PASI and the logRR treatment effect across studies (the PASI 75 response at week 12).

Because some of the included studies also recruited a proportion of non-Caucasian patients, we performed the meta-regression analysis. As shown in Figure 4, the proportion of Caucasian patients was not significantly associated with the efficacy of IL-17 inhibitors (β = 0.3203, *p* = 0.334). We also noticed that the basal PASI seemed to be a little higher in the Asian group, but the meta-regression analysis indicates that the basal PASI was not significantly associated with the efficacy of IL-17 inhibitors (β = −0.0692, *p* = 0.11) (Figure 5).

### Subgroup Analysis

To examine possible effect modification by drug and dose, we performed the subgroup analysis by specific agents and doses (Table 2). For secukinumab 150 and 300 mg, the pooled logRR was approximate between Caucasians and Asians. But, for brodalumab and ixekizumab, there is a difference between Caucasians and Asians.

**Table 2:** Pooled estimates in the subgroup analysis.

**Subgroups**	**Caucasian**				**Asian**			
	**No. of studies**	***I* ^2^**	**Pooled LogRR (95% CI)**	***P***	**No. of studies**	***I* ^**2**^**	**Pooled LogRR (95% CI)**	***P***
SEC 150 mg	6	0.00%	2.78 (2.44, 3.12)	<0.001	4	0.00%	2.82 (2.19, 3.45)	<0.001
SEC 300 mg	3	0.00%	2.86 (2.50, 3.22)	<0.001	4	0.00%	2.96 (2.31, 3.61)	<0.001
BRO 140 mg	2	0.00%	3.22 (2.45, 3.99)	<0.001	1	…	2.30 (1.20, 3.39)	<0.001
BRO 210 mg	2	0.00%	3.50 (2.74, 4.26)	<0.001	1	…	2.48 (1.39, 3.57)	<0.001
IXE 80 mg Q2W	3	70.86%	2.99 (2.34, 3.64)	<0.001	1	…	3.28 (0.55, 6.00)	<0.001
IXE 80 mg Q4W	3	57.94%	2.91 (2.38, 3.45)	<0.001	1	…	3.02 (0.28, 5.76)	<0.001

*RR, risk ratio; SEC, secukinumab; BRO, brodalumab; IXE, ixekinumab; Q4W, every 4 weeks; Q2W, every 2 weeks*.

### Sensitivity Analysis

After removing each independent study and combining the remaining research data, sensitivity analysis was conducted for the meta-analysis. The results were consistent and stable (Supplementary File 4).

## Discussion

### Summary of Evidence

So far, to the best of our knowledge, there is still no clear evidence to clarify the heterogeneity in the efficacy of IL-17 antagonists that is attributable to ethnicity or race. We found out that there is no significant difference in the PASI 75 response between the Asian group and the Caucasian group after a 12-week treatment with IL-17 inhibitor and the meta-regression analysis did not show the association of the proportion of Caucasians with the efficacy of IL-17 inhibitors. For the subgroup analysis, the results of secukinumab 150 and 300 mg showed a consistency of the main analysis. But, for brodalumab and ixekizumab, the result is opposite. In brodalumab, the Caucasian group has better efficacy and in ixekizumab, the Asian group seems a little better. That might be a result from the limited included studies. In Asian RCTs, the basal PASI is slightly higher, which is consistent with the study of Abrouk M. But, the meta-regression analysis did not show association of the basal PASI with the short-term efficacy of IL-17 inhibitors.

The heterogeneity among the included studies was relatively low and might be attributable to the diverse treatment history prior to the enrollment of patients, different-sex ratio, and diverse BMI (some did not provide BMI data). Similarly, methodological differences in study design may also lead to the heterogeneity. For example, the frequency of secukinumab administration is unequal across the studies.

The prevalence of psoriasis is higher in Caucasians than that in Asians and other ethnicities (7, 32). Genome-wide association studies (GWASs) identified multiple psoriasis-associated susceptibility loci, among which HLA-Cw6 was one of the most important alleles in psoriasis (33). The global frequency of the HLA-Cw6 allele varies widely, but is generally higher in Caucasians than in Asians (34, 35).

Of note, three studies (two studies conducted in Italy and one study conducted in Switzerland) showed that HLA-C^*^06:02 was not associated with the response to secukinumab in Caucasians (36–38). Another study conducted in European countries showed that the response to secukinumab and ixekizumab cannot be explained by genetic variation in the *IL-17A* gene (39). However, a pharmacogenomic study by Morelli et al. evaluated the influence of the presence/absence of genetic variants of psoriasis-related loci on the response to secukinumab in Caucasians. They found out that eight single nucleotide polymorphisms (SNPs) in HLA-C and upstream region, including one in HLA-Cw6 and three in *MICB-DT, DDX58*, and *TYK2* genes, were associated with a better response to secukinumab (40). After searching for the National Center of Biotechnology Information database, we noticed that for some SNPs which allele positively associated with the better achieved PAS I75 according to a study by Morelli M, the differences of mutant allele frequency (MAF) between Asians and Caucasians are quite small. For example, Koreans have an MAF of 0.047, and Vietnamese have an MAF of 0.037 in rs4406273, an HLA-Cw6 psoriasis classical allele. Similarly, Northern Swedes have an MAF of 0.045. But, there are also inconsistent findings. The MAF of some SNPs mentioned in a study by Morelli M was different between Asians and Caucasians. Few studies investigated the pharmacogenetics of IL-17A inhibitors in Asians and the MAF data is quite limited. More study is warranted to investigate the genetic factors for IL-17 inhibitors to individualize the treatment.

For the subgroup analysis, we noticed that in brodalumab, the Caucasian group seems to have better efficacy and in ixekizumab, the result seems reverse. Except for the difference in the targets of brodalumab and ixekizumab, *IL-17/IL-17RA* genes polymorphisms which tend to occur in different ethnicity may also contribute to the different response degrees (41, 42). However, it should be pointed out that the number of studies on brodalumab and ixekizumab included in the Asian group was small as well as the sample size. The true efficacy difference of brodalumab and ixekizumab between Asian and White patients remains to be further explored.

In recent years, with the deepening study on the pathogenesis of psoriasis, the important role of interleukin-23 (IL-23)/IL-17 axis in promoting the occurrence and continuation of psoriasis has attracted more and more attention and the studies on the development of IL-23 inhibitors and IL-17 inhibitors and their clinical application are also being carried out continuously. Except for IL-17 monoclonal antibodies, bimekizumab, a new agent targeting IL-17A and IL-17F, has just been reported in phase III clinical trials, showing high efficacy and good safety (43, 44). Since ethnicity-related data were not available for comparison, it was not included in this study.

## Limitations

There are limitations of this study. First, the “Caucasian group” defined in this study also included a small proportion of non-Caucasian patients. Because the individual data were not available, we alternatively used the proportion of Caucasians to perform the meta-regression analysis. Moreover, the Asian group only included Japanese, Indian, and Chinese, while data from other Asian regions, such as Korea, Thailand, and Vietnam, were not available. Therefore, more studies are needed to further identify the differences in the efficacy and safety of IL-17 antagonists between ethnicities. It is also a pity that as far as we know, no statistics of the efficacy of IL-17 inhibitors in Asians living in European and American countries was reported, so we did not analyze the data of these Asians. Besides, the number of included studies was small and the duration of the study was short. For each IL-17 antagonist, the included studies were limited. The rate of the PASI 75 at week 12 was synthesized as the only outcome in this study, since most studies regrouped the patients with different strategies at week 12. The ethnicity-related differences in the long-term efficacy should be tested in real-world settings.

## Conclusion

In summary, based on the currently available data, the short-term efficacy of IL-17 monoclonal antibodies for psoriasis has no significant difference between Caucasians and Asians. Long-term observations in real-world settings in addition to the pharmacogenetic investigations with respect to the ethnicity-related differences in the efficacies of emerging biological agents are warranted for precision medicine.

## Data Availability Statement

The original contributions presented in the study are included in the article/Supplementary Material, further inquiries can be directed to the corresponding author/s.

## Author Contributions

DZ, JQ, MS, and YX involved in the concept and design of this study. DZ did the literature search, performed data analysis, and drafted the manuscript. JQ contributed to the literature search and data analysis. XL edited the tables and figures. DJ and YD critically revised the manuscript. All the authors approved the final version of the manuscript.

## Conflict of Interest

The authors declare that the research was conducted in the absence of any commercial or financial relationships that could be construed as a potential conflict of interest.

## Publisher's Note

All claims expressed in this article are solely those of the authors and do not necessarily represent those of their affiliated organizations, or those of the publisher, the editors and the reviewers. Any product that may be evaluated in this article, or claim that may be made by its manufacturer, is not guaranteed or endorsed by the publisher.

## Supplementary Material

Supplementary File 1 Search terms and strategies.

Supplementary File 2 Supplementary Table 1. Characteristics of included studies. Summary of the design and findings of included studies **(a)** and baseline characteristics of enrolled patient in included studies **(b)**.

Supplementary File 3 Supplementary Figure 1, Supplementary Figure 2, and Supplementary Figure 3.

Supplementary File 4 Sensitivity analysis in the Asian group and the Caucasian group.

Supplementary File 5 The Preferred Reporting Items for Systematic Reviews and Meta-Analyses (PRISMA) checklist.
