# Supplementary material for: Comparison of Efficacy of Anti-interleukin-17 in the Treatment of Psoriasis Between Caucasians and Asians: A Systematic Review and Meta-Analysis
Source: Front Med (Lausanne). 2022 Jan 25;8:814938. doi: 10.3389/fmed.2021.814938 (PMC8822240; doi:10.3389/fmed.2021.814938)
Supplement: Supplementary File 1 — Search terms and strategies. [file Data_Sheet_1.docx]

**Supplementary file 1. Search terms and strategies.**

PUBMED: 306

1. psoriasis

2. parapsoriasis guttata

3. psora

4. Scaly tetter

5. Trichophytia

6. Psoriatic arthritis

7.  1. OR 2. OR 3. OR 4. OR 5. OR 6.

8. Secukinumab OR Cosentyx

9. ixekizumab OR Taltz

10. brodalumab OR Siliq

11.  8. OR 9. OR 10.

12.  randomized controlled trials as topic[MeSH Terms] OR controlled clinical trials as topic[MeSH Terms]

13. randomi* controlled trial* OR randomi* control trial* OR RCT* OR non-randomi* controlled trial* OR non-randomi* control trial* OR controlled clinical trial*

14. randomized[Title/Abstract] OR randomised[Title/Abstract] OR randomly[Title/Abstract]

15. placebo[Title/Abstract]

16. crossover*[Title/Abstract] OR cross over*[Title/Abstract] OR cross-over*[Title/Abstract]

17. "clinical trial"[Publication Type] OR "clinical trials as topic"[MeSH Terms] OR "clinical trial"[All Fields]

18. doubl*[Title/Abstract] OR singl*[Title/Abstract]) AND (blind[Title/Abstract] OR blind*[Title/Abstract]

19.  12. OR 13. OR 14. OR 15. OR 16. OR 17. OR 18.

20.  7. AND 11. AND 19.

EMBASE: 659

(psoriasis or parapsoriasis guttata or trichophytosis ) AND (secukinumab or Cosentyx or ixekizumab or Taltz or brodalumab or Silip) AND (controlled clinical trial or randomized controlled trial ) AND human

THE COCHRANE LIBRARY: 604

1. psoriasis OR psoriasis arthritis (Title Abstract Keyword)
2. secukinumab or Cosentyx or ixekizumab or Taltz or brodalumab or Silip (Title Abstract Keyword)
3. randomi* controlled trial* OR randomi* control trial* OR RCT* (Title Abstract Keyword)

The Wanfang Database, and the Chinese National Knowledge Infrastructure Data of Chinese Journals: 0

“银屑病” AND “（IL-17拮抗剂 OR secukinumab OR ixekizumab OR brodalumab）” AND “随机对照试验”
