# Supplementary material for: Comparison of Efficacy of Anti-interleukin-17 in the Treatment of Psoriasis Between Caucasians and Asians: A Systematic Review and Meta-Analysis
Source: Front Med (Lausanne). 2022 Jan 25;8:814938. doi: 10.3389/fmed.2021.814938 (PMC8822240; doi:10.3389/fmed.2021.814938)
Supplement: Supplementary File 2 — Supplementary Table 1. Characteristics of included studies. Summary of the design and findings of included studies (a) and baseline characteristics of enrolled patient in included studies (b). [file Data_Sheet_2.docx]

| \| **Table S1a.** Summary of the design and findings of included studies \| \| \| \| \| \| \| \| --- \| --- \| --- \| --- \| --- \| --- \| --- \| \| **Study id** \| **Trial** \| **Intervention** \| **Population** \| **Sample size** \| **Outcomes** \| **Adverse events** \| \| Wu NL (2017) \| Phase III (ERASURE) \| Secukinumab vs Placebo \| Taiwanese \| 51 \| PASI 75, PASI 90, PASI 100, IGA at week 12 \| At week 12:  1. Patients with any AE  2. Discontinuation due to AE  3. Non-fatal serious AE \| \| Bhat RM (2017) \| Phase III (FIXTURE) \| Secukinumab vs Placebo \| Indian \| 117 \| PASI 75, PASI 90, PASI 100, IGA, EQ-5D-bsolute mean change, DLQI-ercentage mean change at week 12 \| At week 12:  1. Any Primary system organ class  2. Blood and lymphatic system disorders  3. Infections and infestations  4. Nervous system disorders  5. Skin and subcutaneous tissue disorders \| \| Ohtsuki M (2014) \| Phase III (ERASURE) \| Secukinumab vs Placebo \| Japanese \| 87 \| PASI 75, PASI 90, PASI 100, IGA, response rates, DLQI 0/1 response rates at week 12 \| At week 12:  1. Patients with any AE  2. Treatment emergent serious AE  3. Discontinuation due to AE  4. Most common AE by preferred term \| \| Cai L (2020) \| Phasse III \| Secukinumab vs placebo \| Mainland Chinese \| 441 \| PASI 75, PASI 90, PASI 100, IGA 0/1, DLQI 0/1 response rates at week 12 \| At week 12: 1. Crohn disease \| \| Imafuku S (2017) \| UNCOVER-1 \| Ixekizumab vs Placebo \| Japanese \| 33 \| PASI 75, PASI 90, PASI 100, sPGA (0,1) at week 12 \| At week 12  1. Patients with ≥1 treatment-emergent adverse events  2. TEAE possibly related to study drug 3. Discontinuation because of TEAE  4. Serious adverse event SAE  5. Selected TEAE of special interest \| \| Nakagawa H (2016) \| Phase II \| Brodalumab vs Plaecebo \| Japanese \| 112 \| PASI 75, PASI 90, PASI 100, IGA, DLQI-ercentage mean change at week 12 \| At week 12:  1. Adverse events 2. Common adverse events \| \| Lacour JP (2017) \| JUNCTURE \| Secukinumab vs Placebo \| White proportion:95.05% \| 182 \| PASI 75, PASI 90, PASI 100, IGA 0/1 at week 12 \| N/A \| \| Papp KA (2013) \| Phase II \| Secukinumab vs Placebo \| White proportion:74.29% \| 70 \| PASI 50, PASI 75, PASI 90, IGA at week 12 \| At week 12:  1. Patients with any AE(s)  2. SAEs  3. AEs leading to discontinuation:  4. AEs and infection ≥5% in any treatment group \| \| Rich P (2013) \| Phase II \| Secukinumab vs Placebo \| White proportion:86.98% \| 338 \| PASI 75, PASI 90, IGA 0/1 at week 12 \| At week 12:  1. Any adverse event  2. Serious adverse event  3. Adverse event leading to discontinuation  4. Common adverse events  5. Adverse events of interest \| \| Langley RG (2014) \| Phase III (ERASURE) \| Secukinumab vs Placebo \| White proportion:70.19% \| 738 \| PASI 75, PASI 90, PASI 100 at week 12 \| N/A \| \| Langley RG (2014) \| Phase III (FIXTURE) \| Secukinumab vs Placebo \| White proportion:67.45% \| 980 \| PASI 75, PASI 90, PASI 100 at week 12 \| N/A \| \| Papp KA (2016) \| Phase III (AMAGINE1) \| Brodalumab vs Plaecebo \| White proportion:90.92% \| 661 \| PASI 75, PASI 90, PASI 100 at week 12 \| N/A \| \| Papp KA (2012) \| Phase II \| Brodalumab vs Plaecebo \| White proportion:88.03% \| 117 \| PASI 50, PASI 75, PASI 90, PASI 100 at week 12 \| At week 12:  1. Adverse events 2. Common adverse events \| \| Gordon KB (2016) \| UNCOVER-1 \| Ixekizumab vs Placebo \| White proportion:93.04% \| 1296 \| PASI 75, PASI 90, PASI 100, sPGA Score 0/1 at week 12 \| At week 12:  1. Any adverse event  2. Common adverse event  3. Selected adverse events of special interest  4. Selected serious adverse events of special interest  5. Neutropenia \| \| Griffiths CEM (2015) \| UNCOVER-2 \| Ixekizumab vs Placebo \| White proportion:91.69% \| 866 \| PASI 75, PASI 90, PASI 100, sPGA Score 0/1, DLQI 0/1 at week 12 \| At week 12:  1. Any TEAE  2. Non-fatal serious adverse event  3. Any infection \| \| Griffiths CEM (2015) \| UNCOVER-3 \| Ixekizumab vs Placebo \| White proportion:93.05% \| 964 \| PASI 75, PASI 90, PASI 100, sPGA Score 0/1, DLQI 0/1 at week 12 \| At week 12:  1. Any TEAE  2. Non-fatal serious adverse event  3. Any infection \|   **Abbreviations: PASI 50: achieving a reduction of 50% or more from the baseline in the psoriasis area-and-severity index score; PASI 75: achieving a reduction of 75% or more from the baseline in the psoriasis area-and-severity index score ; PASI 90: achieving a reduction of 90% or more from the baseline in the psoriasis area-and-severity index score ; PASI 100: achieving a reduction of 100% or more from the baseline in the psoriasis area-and-severity index score ; EQ-5D: EuroQol-5 Dimensions; AE:Adverse event; TEAE: Treatment Emergent Adverse Event; sPGA:static Physician's Global Assessment; IGA 0/1: a score of 0 (clear) or 1 (almost clear) on a 5-point modified investigator's global assessment ;Dermatology Life DLQI: Quality Index; N/A：Not mentioned in the study**  **Table S1b.** Baseline characteristics of enrolled patients in included studies | | | | | | |
| --- | --- | --- | --- | --- | --- | --- | --- | --- | --- | --- | --- | --- | --- | --- | --- | --- | --- | --- | --- | --- | --- | --- | --- | --- | --- | --- | --- | --- | --- | --- | --- | --- | --- | --- | --- | --- | --- | --- | --- | --- | --- | --- | --- | --- | --- | --- | --- | --- | --- | --- | --- | --- | --- | --- | --- | --- | --- | --- | --- | --- | --- | --- | --- | --- | --- | --- | --- | --- | --- | --- | --- | --- | --- | --- | --- | --- | --- | --- | --- | --- | --- | --- | --- | --- | --- | --- | --- | --- | --- | --- | --- | --- | --- | --- | --- | --- | --- | --- | --- | --- | --- | --- | --- | --- | --- | --- | --- | --- | --- | --- | --- | --- | --- | --- | --- | --- | --- | --- | --- | --- | --- | --- | --- | --- | --- | --- | --- | --- | --- | --- | --- | --- |
| **Study id** | **Drugs and Doses** | **Age**  **(years, mean±SD)** | **Gender composition (male: female)** | **BMI**  **(mean±SD)** | **PASI scores (mean±SD)** | **Previous treatment history** |
| Wu NL (2017) | Placebo | 40.6±10.8 | 13:2 | 27.9±6.3 | 21.1±6.5 | 1. Previous systemic treatment:  Any:14(93.3%)  Conventional agent:12(80%) Phototherapy:11(73.3%)  2. Biologics:  TNF inhibitors:1(6.7%)  Anti-IL-12/IL-23 p40 agent:2(13.3%)  Other biologics:1(6.7%) |
|  | Secukinumab 150mg (once weekly at weeks 0, 1, 2, 3 and 4, and then every 4 weeks) | 39.5±10.9 | 14:6 | 26.0±4.2 | 20.9±7.7 | 1. Previous systemic treatment:  Any:17(85%)  Conventional agent:15(75%) Phototherapy:12(60%)  2. Biologics:  TNF inhibitors:5(25%)  Anti-IL-12/IL-23 p40 agent:5(25%)  Other biologics:1(5%) |
|  | Secukinumab 300mg (once weekly at weeks 0, 1, 2, 3 and 4, and then every 4 weeks) | 38.1±12.0 | 14:2 | 26.7±3.8 | 24.7±8.5 | 1. Previous systemic treatment:  Any:14(87.5%)  Conventional agent:12(75%) Phototherapy:11(68.8%)  2. Biologics:  TNF inhibitors:4(25%)  Anti-IL-12/IL-23 p40 agent:2(12.5%)  Other biologics:2(12.5%) |
| Bhat RM (2017) | placebo | 42.3±13.7 | 29:14 | 25.75±4.959 | 28.00±11.5001 | 1. Previous exposure to systemic psoriasis therapy:40(93.0%)  2. Previous failure to systemic psoriasis therapy:40(93.0%) |
|  | Secukinumab 150mg (at weeks 0,1,2,3,4,8,12) | 45.0±11.94 | 29:5 | 25.68±4.462 | 28.16±12.7079 | 1. Previous exposure to systemic psoriasis therapy:31(91.2%)  2. Previous failure to systemic psoriasis therapy:30(88.2%) |
|  | Secukinumab 300mg (at weeks 0,1,2,3,4,8,12) | 43.7±13.94 | 28:13 | 24.32±4.122 | 28.04±10.2637 | 1. Previous exposure to systemic psoriasis therapy:35(85.4%)  2. Previous failure to systemic psoriasis therapy:33(80.5%) |
| Ohtsuki M (2014) | Placebo (once weekly at baseline and at weeks 1, 2, 3 and 4, then every 4 weeks) | 50.2±13.62 | 23:6 | 25.8±5.16 | 21.4±10.31 | 1. Phototherapy Previous systemic treatment:12(41.4%) 2. Any:20(69.0%) 3. Biologic:6(20.7%) 4. Anti-p40:2(6.9%) 5. Anti-TNF:5(17.2%) |
|  | Secukinumab 150mg (once weekly at baseline and at weeks 1, 2, 3 and 4, then every 4 weeks) | 48.2±13.08 | 23:6 | 26.5±4.41 | 28.2±13.64 | 1. Phototherapy Previous systemic treatment:10(34.5%) 2. Any:21(72.4%) 3. Biologic:5(17.2%) 4. Anti-p40:3(10.3%) 5. Anti-TNF:3(10.3%) |
|  | Secukinumab 300mg (once weekly at baseline and at weeks 1, 2, 3 and 4, then every 4 weeks) | 51.9±11.77 | 26:3 | 26.9±3.78 | 26.7±10.49 | 1. Phototherapy Previous systemic treatment:11(37.9%) 2. Any:25(86.2%) 3. Biologic:6(20.7%) 4. Anti-p40:5(17.2%) 5. Anti-TNF:1(3.4%) |
| Cai L (2020) | placebo | 38.7±10.3 | 89:21 | N/A | 26.2±9.3 | 1. Previous exposure to biologic psoriasis therapy:23(20.9%) |
|  | Secukinumab 150mg (at weeks 1,2,3,4,8,12) | 40.5±10.8 | 84:26 | N/A | 26.5±10.6 | 1. Previous exposure to biologic psoriasis therapy:24(21.8%) |
|  | Secukinumab 300mg (at weeks 1,2,3,4,8,12) | 39.0±11.6 | 177:54 | N/A | 27.3±10.9 | 1. Previous exposure to biologic psoriasis therapy:33(14.9%) |
| Imafuku S (2017) | Placebo | 51.4±14.9 | 9:4 | N/A | 24.8±12.9 | 1. Previous systemic therapy:  2. Non-biological only:4(38.8%)  3. Biological only:0  4. Non-biological + biological:5(35.5%)  5. Never used:4(30.8%) |
|  | Ixekizumab 80mg every 4 weeks | 44.5±10.6 | 10:2 | N/A | 22.3±9.4 | 1. Previous systemic therapy:  2. Non-biological only:7(58.3%)  3. Biological only:0  4. Non-biological + biological:2(16.7%)  5. Never used:3(25.0%) |
|  | Ixekizumab 80mg every 2 weeks | 45.5±10.4 | 8:0 | N/A | 27.6±14.7 | 1. Previous systemic therapy:  2. Non-biological only:5(62.5%)  3. Biological only:0  4. Non-biological + biological:1(12.5%)  5. Never used:2(25.0%) |
| Nakagawa H (2016) | Placebo (injected subcutaneously at baseline and weeks 1, 2, 4, 6, 8) | 46.6±10.8 | 27:11 | 26.02±4.68 | 23.97±8.90 | 1. Prior systemic or photo therapies:34(89.5%)  2. Prior biologic treatment:3(7.9%) |
|  | Brodalumab 140mg (injected subcutaneously at baseline and weeks 1, 2, 4, 6, 8) | 46.4±13.2 | 30:7 | 26.26±4.84 | 28.53±10.77 | 1. Prior systemic or photo therapies:30(81.1%)  2. Prior biologic treatment:3(8.1%) |
|  | Brodalumab 210mg (injected subcutaneously at baseline and weeks 1, 2, 4, 6, 8) | 46.4±11.8 | 29:8 | 26.34±5.63 | 27.98±14.35 | 1. Prior systemic or photo therapies:28(75.7%)  2. Prior biologic treatment:5(13.5%) |
| Lacour JP (2017) | Secukinumab 300mg subcutaneous at baseline, weeks 1, 2 and 3 and then every 4 weeks from week 4 to week 12 | 46.6±14.23 | 46:14 | 30.0±6.90 | 18.9±6.37 | 1. Systemic therapy:34(56.7%) 2. Conventional systemic agents:30(50.0%) 3. Biologic systemic agents:15(25.0%) |
|  | Secukinumab 150mg subcutaneous at baseline, weeks 1, 2 and 3 and then every 4 weeks from week 4 to week 12 | 43.9±14.41 | 41:20 | 30.6±9.50 | 22.0±8.85 | 1. Systemic therapy:34(55.7%) 2. Conventional systemic agents:31(50.8%) 3. Biologic systemic agents:15(24.6%) |
|  | Placebo | 43.7±12.74 | 38:23 | 30.0±6.82 | 19.4±6.70 | 1. Systemic therapy:33(54.1%) 2. Conventional systemic agents:29(47.5%) 3. Biologic systemic agents:13(21.3%) |
| Papp KA (2013) | Secukinumab 3*75mg each month | 45.8±12.36 | 14:07 | N/A | 19.7±6.88 | 1. UV therapy:13(61.9%)  2. Systemic therapy:11(52.4%) 3. Biologic therapy:4(19.0%) |
|  | Secukinumab 3*150mg each month | 45.4±11.46 | 21:06 | N/A | 21.3±9.41 | 1. UV therapy:18(66.7%);  2. Systemic therapy:15(55.6%); 3. Biologic therapy:8(29.6%) |
|  | Placebo | 45.9±10.88 | 14:08 | N/A | 21.7±8.53 | 1. UV therapy:16(72.7%);  2. Systemic therapy:16(72.7%); 3. Biologic therapy:8(36.4%) |
| Rich P (2013) | Secukinumab 150mg at week 0,4 and 8 | 44.2±12.96 | 104:34 | N/A | 20.8±8.08 | 1. UV therapy:93(67.4%)  2. Systemic therapy:109(79%)  3. Biologic therapy:41(29.7%) |
|  | Secukinumab 150mg at week 0,1,2 and 4 | 44.5±12.45 | 105:28 | N/A | 19.9±7.81 | 1. UV therapy:88(66.2%)  2. Systemic therapy:98(73.7%)  3. Biologic therapy:40(30.1%) |
|  | Placebo | 44.2±12.59 | 44:23 | N/A | 20.5±9.31 | 1. UV therapy:40(59.7%)  2. Systemic therapy:42(62.7%)  3. Biologic therapy:17(25.4%) |
| Langley RG (ERASURE)(2014) | Secukinumab 300mg (once  weekly at baseline and at weeks 1, 2, 3, and 4  and then every 4 weeks) | 44.9±13.5 | 169:76 | 30.3±7.2 | 22.5±9.2 | 1. Previous systemic treatment:  Any:163(66.5%)  Conventional agents:128(52.2%) Biologic agents:70(28.6%) 2. TNF inhibitor:48(19.6%) 3. Anti-interleukin-12 and anti-interleukin-23 agent:32(13.1%) |
|  | Secukinumab 150mg (once  weekly at baseline and at weeks 1, 2, 3, and 4  and then every 4 weeks) | 44.9±13.3 | 168:77 | 29.8±6.8 | 22.3±9.8 | 1. Previous systemic treatment:  Any:156(63.7%) Conventional agents:125(51.0%)  Biologic agents:73(29.8%) 2. TNF inhibitor:44(18.0%) 3. Anti-interleukin-12 and anti-interleukin-23 agent:37(15.1%) |
|  | placebo | 45.4±12.6 | 172:76 | 30.3±7.8 | 21.4±9.1 | 1. Previous systemic treatment:  Any:146(58.9%)  Conventional agents:108(43.5%)  Biologic agents:73(29.4%) 2. TNF inhibitor:51(20.6%) 3. Anti-interleukin-12 and anti-interleukin-23 agent:31(12.5%) |
| Langley RG (FIXTURE) (2014) | Secukinumab 300mg (once  weekly at baseline and at weeks 1, 2, 3, and 4  and then every 4 weeks) | 44.5±13.2 | 224:103 | 28.4±6.4 | 23.9±9.9 | 1. Previous systemic treatment:  Any:206(63.0%)  Conventional agents:195(59.6%)  Biologic agents:38(11.6%) 2. TNF inhibitor:12(3.0%) 3. Anti-interleukin-12 and anti-interleukin-23 agent:23(7.0%) |
|  | Secukinumab 150mg (once  weekly at baseline and at weeks 1, 2, 3, and 4  and then every 4 weeks) | 45.4±12.9 | 236:91 | 28.4±5.9 | 23.7±10.5 | 1. Previous systemic treatment:  Any:212(64.8%)  Conventional agents:198(60.6%)  Biologic agents:45(13.8%) 2. TNF inhibitor:15(4.6%) 3. Anti-interleukin-12 and anti-interleukin-23 agent:23(7.0%) |
|  | Placebo | 44.1±12.6 | 237:89 | 27.9±6.1 | 24.1±10.5 | 1. Previous systemic treatment:  Any:204(62.6%)  Conventional agents:199(61.0%)  Biologic agents:35(10.7%) 2. TNF inhibitor:12(3.7%) 3. Anti-interleukin-12 and anti-interleukin-23 agent:21(6.4%) |
| Papp KA (2016) | Placebo | 47±13 | 161:59 | 30.3±6.6 | 19.7±7.7 | N/A |
|  | Brodalumab 140mg every 2 weeks | 46±13 | 162:57 | 30.5±6.9 | 20.0±7.4 | N/A |
|  | Brodalumab 210mg every 2 weeks | 46±12 | 161:61 | 31.0±7.7 | 19.4±6.6 | N/A |
| Papp KA (2012) | Placebo | 41.8±14.4 | 22:11 | 29.3±6.8 | 18.9±5.9 | 1. Prior topical treatment:34(89%)  2. Prior phototherapy:  PUVA:7(18%) UVB:17(45%) 3. Prior systemic therapy:27(71%)  4. Nonbiologic therapy: Methotrexate:9(24%)  Oral or intramuscular glucocorticoids:5(13%) Cyclosporine:3(8%)  Oral retinoids:2(5%)  Biologic therapy:  Etanercept:7(18%)  Adalimumab:4(11%) Ustekinumab:5(13%) |
|  | Brodalumab 140mg(on day 1 and at weeks 1, 2, 4, 6, 8, and 10) | 44.0±11.7 | 28:11 | 30.8±7.0 | 19.4±8.0 | 1. Prior topical treatment:39(100%)  2. Prior phototherapy:  PUVA:8(21%)  UVB:12(31%) 3. Prior systemic therapy:25(64%) 4. Nonbiologic therapy: Methotrexate:16(41%) Oral or intramuscular glucocorticoids:3(8%) Cyclosporine:4(10%) Oral retinoids:6(15%)  Biologic therapy:  Etanercept:3(8%)  Adalimumab:5(13%)  Ustekinumab:2(5%) |
|  | Brodalumab 210mg(on day 1 and at weeks 1, 2, 4, 6, 8, and 10) | 42.1±12.2 | 25:15 | 29.8±6.6 | 20.6±7.8 | 1. Prior topical treatment:37(92%)  2. Prior phototherapy:  PUVA:8(20%)  UVB:15(38%) 3. Prior systemic therapy:32(80%)  4. Monbiologic therapy: Methotrexate:16(40%)  Oral or intramuscular glucocorticoids:4(10%) Cyclosporine:4(10%) Oral retinoids:7(18%)  Biologic therapy:  Etanercept:4(10%) Adalimumab:7(18%) Ustekinumab:6(15%) |
| Gordon KB (2016) | Placebo | 46±13 | 303:128 | N/A | 20±9 | 1. Phototherapy:185(42.9%)  2. Nonbiologic systemic:224(52.0%) 3. Biologic:181(42.0%) |
|  | Ixekizumab 80mg every 4 weeks after a starting dose of 160mg | 46±13 | 289:143 | N/A | 20±7 | 1. Phototherapy:205(47.5%)  2. Nonbiologic systemic:213(49.3%) 3. Biologic:168(38.9%) |
|  | Ixekizumab 80mg every 2 weeks after a starting dose of 160mg | 45±12 | 291:142 | N/A | 20±8 | 1. Phototherapy:201(46.4%)  2. Nonbiologic systemic:247(57.0%) 3. Biologic:173(40.0%) |
| Griffiths CEM (UNCOVER-2) (2015) | Placebo 160mg starting does followed by 80mg every 2 weeks | 45±12 | 120:48 | 31±7 | 21±8 | 1. Topical prescription:142(85%)  2. Phototherapy:74(44%)  3. Non-biological systemic:80(48%)  4. Biological:43(26%) |
|  | Ixekizumab 160mg starting does followed by 80mg every 4 weeks | 45±14 | 244:103 | 31±7 | 20±7 | 1. Topical prescription:289(83%)  2. Phototherapy:160(46%)  3. Non-biological systemic:178(51%)  4. Biological:85(25%) |
|  | Ixekizumab 160mg starting does followed by 80mg every 2 weeks | 45±13 | 221:130 | 30±7 | 19±7 | 1. Topical prescription:289(82%)  2. Phototherapy:163(46%)  3. Non-biological systemic:178(51%)  4. Biological:84(24%) |
| Griffiths CEM (UNCOVER-3)(2015) | Placebo 160mg starting does followed by 80mg every 2 weeks | 46±12 | 137:56 | 30±6 | 21±8 | 1. Topical prescription:147(76%)  2. Phototherapy:60(31%)  3. Non-biological systemic:82(43%)  4. Biological:33(17%) |
|  | Ixekizumab 160mg starting does followed by 80mg every 4 weeks | 46±13 | 258:128 | 31±7 | 21±8 | 1. Topical prescription:303(79%)  2. Phototherapy:154(40%)  3. Non-biological systemic:181(47%)  4. Biological:58(15%) |
|  | Ixekizumab 160mg starting does followed by 80mg every 2 weeks | 46±13 | 254:131 | 30±7 | 21±8 | 1. Topical prescription:303(79%)  2. Phototherapy:151(39%)  3. Non-biological systemic:170(44%)  4. Biological:58(15%) |

**Abbreviations: N/A:Not mentioned in the study; UVB: ultraviolet radiation B; PUVA: Psoralen and ultraviolet radiation A therapy； UV therapy: ultraviolet radiation therapy.**
