# Supplementary material for: Comparison of Efficacy of Anti-interleukin-17 in the Treatment of Psoriasis Between Caucasians and Asians: A Systematic Review and Meta-Analysis
Source: Front Med (Lausanne). 2022 Jan 25;8:814938. doi: 10.3389/fmed.2021.814938 (PMC8822240; doi:10.3389/fmed.2021.814938)
Supplement: Supplementary File 3 — Supplementary Figure 1, Supplementary Figure 2, and Supplementary Figure 3. [file Data_Sheet_3.docx]

**Fig S1:** Risk of bias graph: authors' judgements about each risk of bias item presented as percentages across all involved publications.**
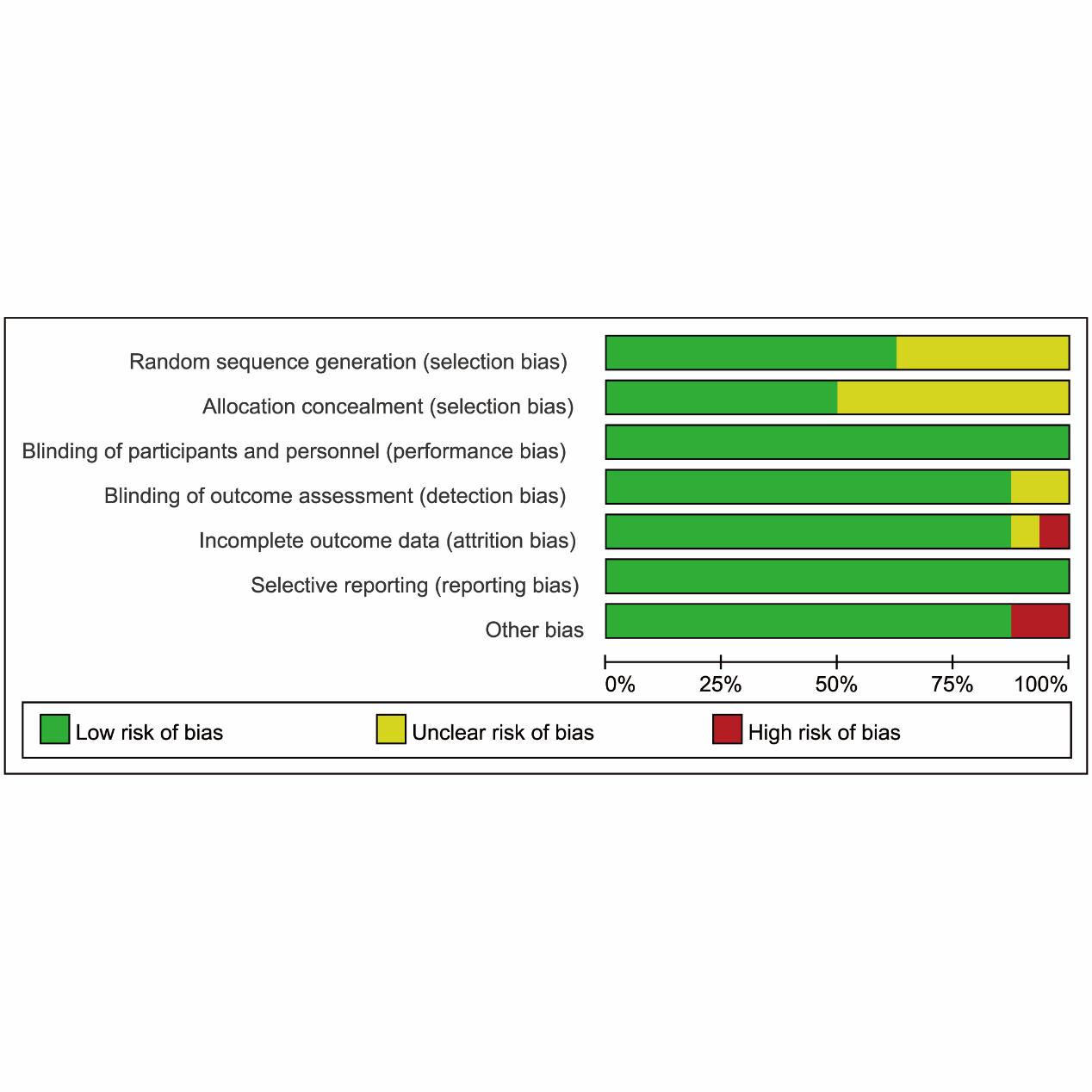
**

**Fig. S2.** Risk of bias summary for individual studies.


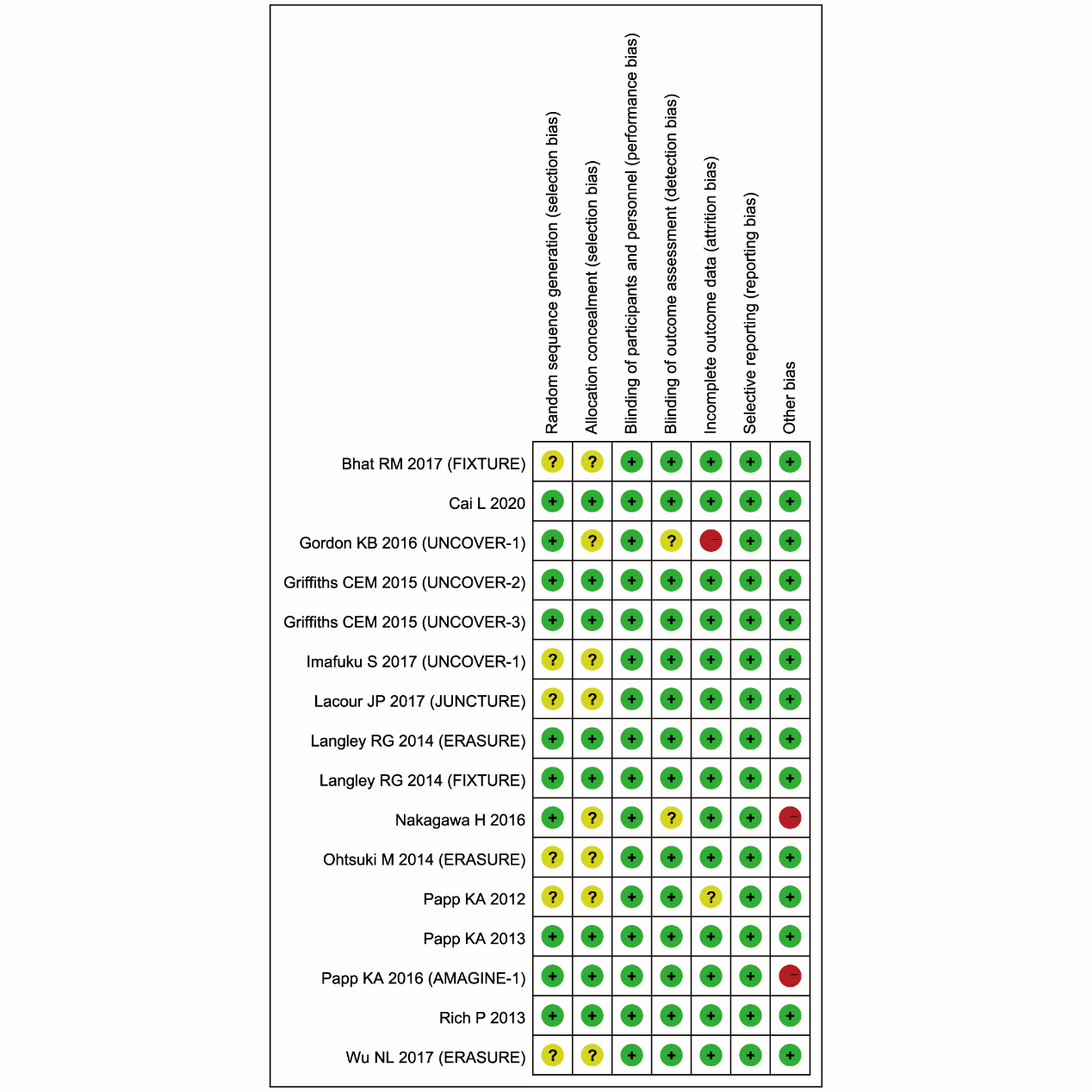


**Fig. S3.** Funnel plots with pseudo 95% confidence limits.

**
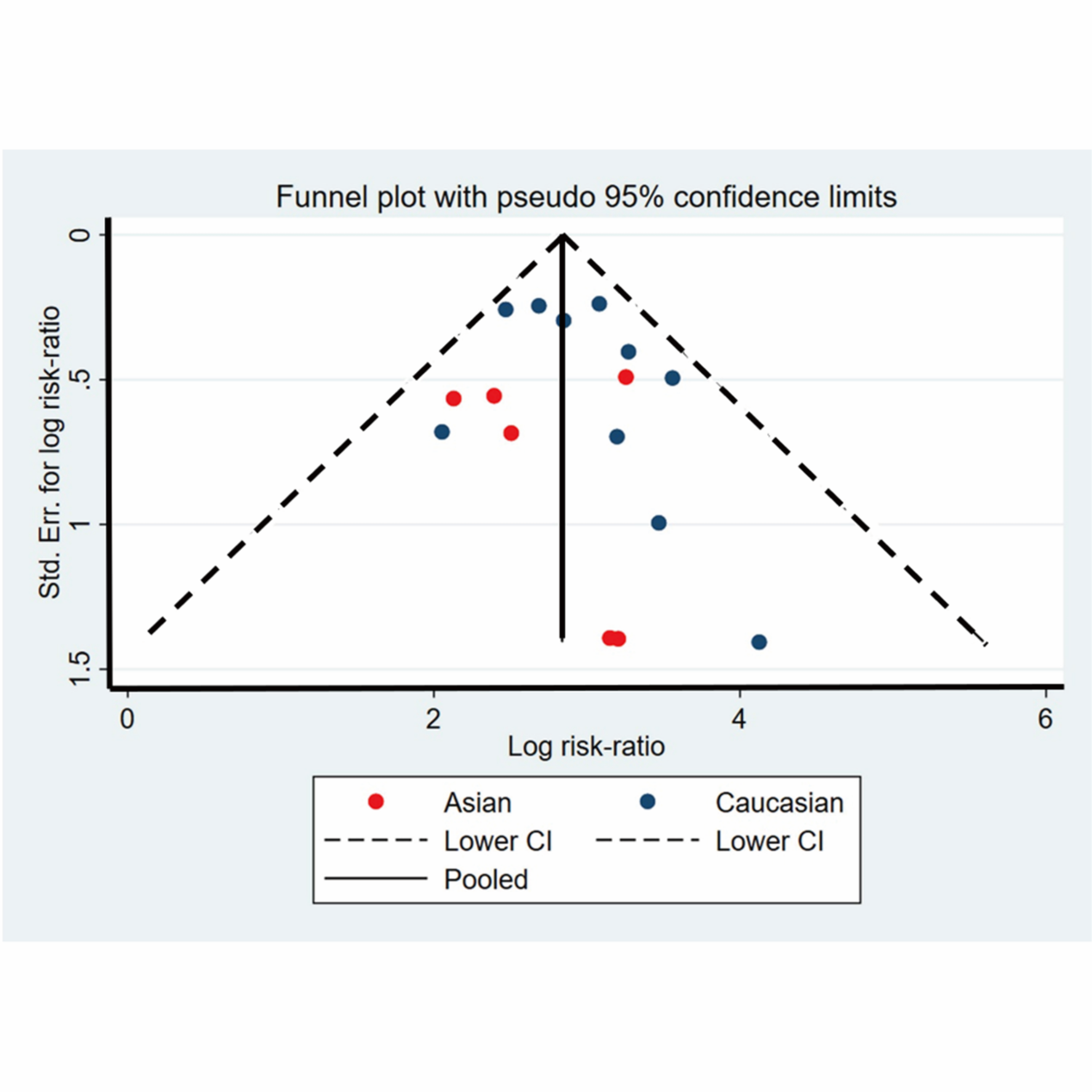
**

The red dots represent Asian studies and the dark blue dots represent Caucasian studies. CI=confidence interval.
