# Supplementary material for: Comparison of Efficacy of Anti-interleukin-17 in the Treatment of Psoriasis Between Caucasians and Asians: A Systematic Review and Meta-Analysis
Source: Front Med (Lausanne). 2022 Jan 25;8:814938. doi: 10.3389/fmed.2021.814938 (PMC8822240; doi:10.3389/fmed.2021.814938)
Supplement: Supplementary File 4 — Sensitivity analysis in the Asian group and the Caucasian group. [file Data_Sheet_4.docx]

**Sensitivity analysis in Asian group and Caucasian group.**

**Asian:**

**Caucasian:**
