# Supplementary material for: Comparison of Efficacy of Anti-interleukin-17 in the Treatment of Psoriasis Between Caucasians and Asians: A Systematic Review and Meta-Analysis
Source: Front Med (Lausanne). 2022 Jan 25;8:814938. doi: 10.3389/fmed.2021.814938 (PMC8822240; doi:10.3389/fmed.2021.814938)
Supplement: Supplementary File 6 — Supplementary Methods. [file Data_Sheet_6.pdf]

Supplementary methods

Discrepancies between the registered protocol and the final manuscript

In this meta-analysis, the control group was only patients who received a placebo, the PASI 75 was chosen as the main outcome, and the measure of effect was a relative risk. These adjustments are based on the common characteristics of the collected data. Although we still wanted to study some of the problems mentioned in the registered protocol, it was not implemented due to the lack of data.
